# The Development of a 3D Printer-Inspired, Microgravity-Compatible Sample Preparation Device for Future Use Inside the International Space Station

**DOI:** 10.3390/mi14050937

**Published:** 2023-04-26

**Authors:** Kamfai Chan, Arunkumar Arumugam, Cole Markham, Ryan Jenson, Hao-Wei Wu, Season Wong

**Affiliations:** 1 AI Biosciences, Inc., College Station, TX 77845, USA; 2 IRPI, LLC, Wilsonville, OR 97070, USA

**Keywords:** DNA extraction, nucleic acid purification, microgravity compatibility, molecular detection, automated sample preparation

## Abstract

Biological testing on the International Space Station (ISS) is necessary in order to monitor the microbial burden and identify risks to crew health. With support from a NASA Phase I Small Business Innovative Research contract, we have developed a compact prototype of a microgravity-compatible, automated versatile sample preparation platform (VSPP). The VSPP was built by modifying entry-level 3D printers that cost USD 200–USD 800. In addition, 3D printing was also used to prototype microgravity-compatible reagent wells and cartridges. The VSPP’s primary function would enable NASA to rapidly identify microorganisms that could affect crew safety. It has the potential to process samples from various sample matrices (swab, potable water, blood, urine, etc.), thus yielding high-quality nucleic acids for downstream molecular detection and identification in a closed-cartridge system. When fully developed and validated in microgravity environments, this highly automated system will allow labor-intensive and time-consuming processes to be carried out via a turnkey, closed system using prefilled cartridges and magnetic particle-based chemistries. This manuscript demonstrates that the VSPP can extract high-quality nucleic acids from urine (Zika viral RNA) and whole blood (human RNase P gene) in a ground-level laboratory setting using nucleic acid-binding magnetic particles. The viral RNA detection data showed that the VSPP can process contrived urine samples at clinically relevant levels (as low as 50 PFU/extraction). The extraction of human DNA from eight replicate samples showed that the DNA extraction yield is highly consistent (there was a standard deviation of 0.4 threshold cycle when the extracted and purified DNA was tested via real-time polymerase chain reaction). Additionally, the VSPP underwent 2.1 s drop tower microgravity tests to determine if its components are compatible for use in microgravity. Our findings will aid future research in adapting extraction well geometry for 1 g and low g working environments operated by the VSPP. Future microgravity testing of the VSPP in the parabolic flights and in the ISS is planned.

## 1. Introduction

Monitoring microbial burden is one of NASA’s environmental monitoring criteria for identifying risks to crew health during missions [1,2,3,4,5,6,7,8,9,10,11]. Currently, biological testing on the International Space Station (ISS) is limited to the available upmass, downmass, crew time, and preexisting interface and hardware capabilities [12]. Other factors include reliance on the return sample and ground analysis, and equipment constraints regarding size, mass, power, lack of portability, and insufficient calibration life.

An automated sample collection, preparation, and possible analysis system could simplify the complex sample preparation steps that are often carried out manually and alleviate some of these issues. This article will describe our efforts to develop and test a small-footprint, robust, gravity-independent, and closed sample preparation system. To our knowledge, there are currently no closed and compact turnkey systems for sample preparation in space. The possibility of carrying out sample preparation in an enclosed format would enable sample preparation for molecular biology tests from various sample matrices (cell culture, plant tissue, blood, swab, etc.) without the risk of cross-contamination. By coupling our device with suitable downstream detection modules, NASA can expand its capabilities to include processes such as polymerase chain reaction (PCR), isothermal amplification, and even sequencing [13,14,15]. With the development of additional assays, new applications could be extended to include microbial detection in advanced life support systems, in vitro testing for infectious diseases during space flight, gene expression analysis for radiation exposure monitoring/studies, and testing the effect of space flights on microbial gene expression and virulence. By offering a medium-throughput, magnetic particle-based sample preparation platform, our versatile sample preparation platform (VSPP) unit will enable NASA-supported researchers and private companies to carry out in situ life sciences research and commercial projects inside the ISS with near real-time result capability. It is important to note that the National Center for Advancing Translational Sciences of US NIH and NASA created the “Tissue Chips in Space initiative” in 2016 in order to better understand the role of microgravity in human health and disease [16]. They have supported the building of automated platforms; however, these systems are usually very specialized and not flexible enough to perform other tasks.

Compared with standard manual spin-column-based separation procedures, magnetic particle-based separation techniques have several advantages, including simplicity in handling and great potential for automation [17,18,19,20,21]. Magnetic particle-based methods are well-characterized and effective in extracting highly purified nucleic acids for subsequent analysis and are consequently employed in many extraction kits. Additionally, magnetic particle-based purification has also been developed for proteins and extraction kits have a shelf life of at least 18 to 24 months when stored at 8 °C. Unfortunately, most commercially available magnetic particle-based automated sample preparation devices are complicated to use and expensive to purchase and maintain. Most importantly, they are not designed for use in microgravity. By combining magnetic particle-based extraction technology with low-cost hardware and cartridges, we developed the VSPP, which we are currently validating with the eventual goal of certifying it for use in space.

The VSPP was inspired by the commercialization of consumer-grade 3D printers with multipurpose features. In the last decade, the 3D printer market has expanded rapidly after a key patent on fused deposition modeling (FDM) expired. Thus, they can be purchased at increasingly affordable prices (USD 200 to USD 800) with improved quality and speed. As discussed in our prior publications, there are several features of FDM-based 3D printers that have allowed us to repurpose them as nucleic acid extraction devices with the ability to perform nucleic acid amplification [22,23,24,25]. In addition to repurposing 3D printers into sample preparation devices, we have utilized 3D printing technology to design and prototype specially shaped, microgravity-compatible wells for sample preparation procedures to be performed inside the ISS.

**Figure 1:**
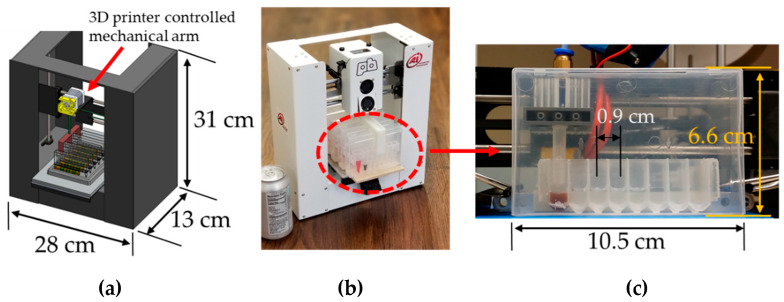
Converted 3D printer units: (**a**) A schematic 3D printer (Printrbot Play) converted into a sample preparation device. The printer can hold up to six cartridges (also shown in Figure 2). (**b**) An actual unit that was developed. A soda can is shown as a size reference. (**c**) Magnetic coupling paired with the 3D printer’s motion control is used to perform extraction procedures and controlled from the outside of an enclosed cartridge. The extraction tip’s movements are controlled via magnetic coupling through the side wall. There is no direct contact between the instrument and the cartridge’s contents. The size of the cartridge box is 66 × 105 × 15 mm^3^. An 8-well strip cut from a standard 96-well extraction plate was used to store extraction reagent buffer solutions in (**c**). The spacing between the wells is 9 mm.

We have previously used different repurposed 3D printers to perform DNA and RNA extraction in 1 g conditions, obtaining excellent results compared to standard methods requiring manual labor, such as spin-column methods (Figure 1) [22,23,26]. The extracted nucleic acids captured on magnetic particles can be eluted into an elution buffer (e.g., Tris-HCl or water) or directly into master mix. In space, nucleic acids can be eluted into a Cepheid SmartCycler (already installed inside the ISS) PCR reaction tube to perform real-time PCR [8,27,28]. For space applications, the lyophilization of PCR and isothermal amplification reagents means that the preloaded reaction mix can be stored for months without refrigeration. Alternatively, assay reagents and remaining templates could be stored in NASA’s −80 °C freezer onboard the ISS.

DNA/RNA-based analysis in biodetection relies heavily on the success of nucleic acid extraction from complex sample matrixes. Upstream sample preparation steps require the user to effectively lyse cells, recover and purify nucleic acids by removing interfering contaminants from samples.

### Design of an Extraction Cartridge for Use in Microgravity

Although we have not had cross-contamination issues from nucleic acid extraction and elution in an open-environment format when using the VSPP in a 1 g environment, using the VSPP in microgravity will require an enclosed cartridge during extraction because droplets are more likely to escape from the surface of the reagents and travel to neighboring wells. Therefore, we used a previously developed self-contained cartridge for extraction in microgravity [29].

**Figure 2:**
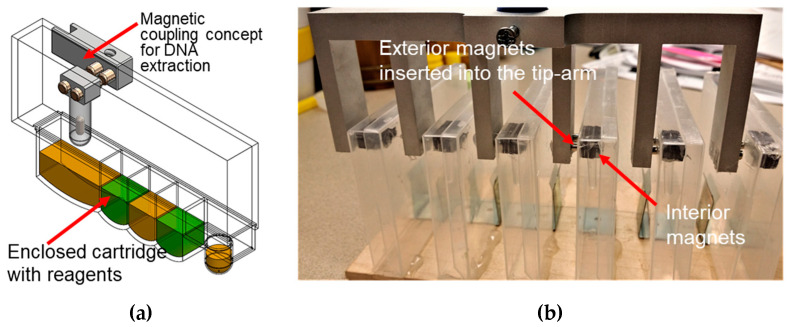
Magnetic coupling concept: (**a**) schematic view of one cartridge showing the use of magnetic coupling actuation; (**b**) view showing the magnetic tips inside the cartridges, connected to magnets outside of the cartridge. The movement of the exterior magnets indirectly controls the magnetic tips in the cartridge interior. The stepper motors on a repurposed 3D printer can be programmed to automate mechanical movements that control the magnets to manipulate other components (e.g., extraction tips) inside the cartridge to successfully perform nucleic acid extraction.

**Figure 3:**
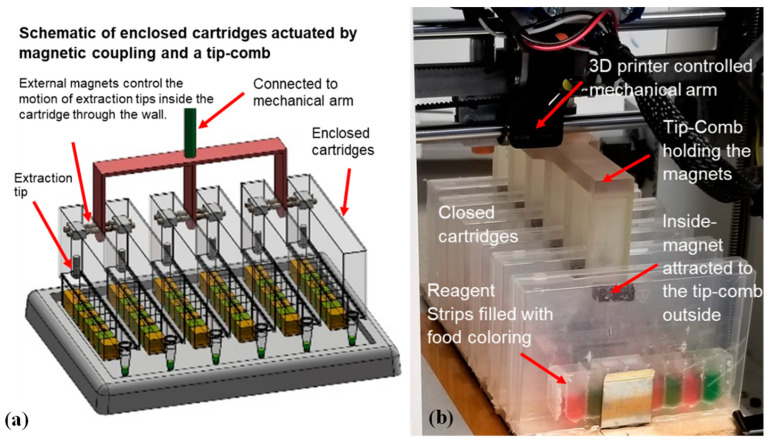
VSPP with multiple cartridges: (**a**) a drawing of the use of a tip-comb and magnetic coupling to process multiple self-contained cartridges simultaneously; (**b**) a photo of the tip-comb actuating the procedure for extraction inside of six prototype cartridges. The wells were filled with food-coloring solutions for more clarity. The extraction tips’ movements are controlled via magnetic coupling through the side wall.

Magnets are coupled on the interior and exterior of the cartridge wall so that the movement of the exterior magnets controls the movements of the interior magnets (Figure 2). Programming the stepper motors on a repurposed 3D printer can fully automate the mechanical movements needed for nucleic acid extraction, even in an enclosed environment. In this design, no direct contact occurs between the instrument and the cartridge’s contents. The VSPP can also perform nucleic acid extraction with multiple cartridges simultaneously using a tip-comb connected to multiple sets of magnetic coupling magnets (Figure 3). The magnets also facilitate the automatic self-alignment of the actuator tips within the cartridge, regardless of position changes due to the microgravity environment, helping to translate our device for use in microgravity. A detailed explanation of the cartridge’s operation has been presented previously [29]. We note that to improve the yield and shorten the elution time, a heating strip under the elution well (not shown) was used in some of the experiments presented.

Other NASA undertakings include pipetting a DNA library into the MinION sequencer (Oxford Nanopore Technologies) in space, a process carried out by biochemist and astronaut Kate Rubins during Expedition 49 in 2016 [30,31,32,33]. This demonstrated that manual pipetting steps and DNA sequencing are compatible with a microgravity environment. Next, we aim to demonstrate that our novel approach for automated processes will have a high likelihood of success when fully developed for use in space, reducing crew time and enabling a higher throughput. As we are still in the early stages of the VSPP’s development, we were limited to initial tests on the compatibility of our device in microgravity in the limited time available (two seconds) during the drop tower tests. However, our next steps are to test the entire system in settings that offer an extended period of microgravity or reduced gravity (e.g., parabolic flights or suborbital flights).

## 2. Materials and Methods

### 2.1. Using Magnetic Coupling to Extract Nucleic Acids Inside an Enclosed Cartridge

Individually enclosed environments are primarily used to prevent cross-contamination, which is critical to avoid in molecular diagnostics. However, it is particularly essential to prevent any release of potentially harmful microbes and extraction reagents in low g environments such as the ISS. We used prefilled reagent strips, sealed with aluminum foil, which were placed inside a strip holder that can be closed after sample input. Expanding this design into a magnetic tip-comb allows for the parallel processing of multiple cartridges (Figure 3), as described previously [24,29]. We tested different actuation speeds with the tip-comb to perform extraction via magnetic coupling in order to determine the maximum speed possible to minimize the test time without dislodging the magnets. While the VSPP’s operating speed is not critical when performing research work inside the ISS, we performed this test to show the robustness and stability of the magnetic coupling actuation mechanism.

### 2.2. Zika Virus and Urine Specimens

ZIKV (a 2015 strain from Mexico) was harvested from Vero cells four days post-infection at a concentration of approximately 2 × 10^7^ plaque-forming units (PFU)/mL. The ZIKV suspension was then mixed with three volumes of TRIzol Reagent (Thermo Fisher Scientific [TFS], Waltham, MA, USA) to bring the concentration of ZIKV to 5 × 10^6^ PFU/mL. Deidentified normal human urine specimens (Catalog# IR100007P) were purchased from Innovative Research (Novi, MI, USA). These samples were extracted using previously developed protocols [23]. The commercially purchased urine was not subject to any institutional IRB according to USA regulations.

### 2.3. RNA Isolation for Pathogen Detection

Two sets of experiments using Zika virus RNA in urine were set up using identical sample concentrations and volumes for extraction. One set of experiments was performed manually, while the other set of experiments was performed using our 3D printer-based VSPP (built from the framework of a Printrbot Play platform). To test the VSPP’s range, the Zika virus culture was diluted to 10X, 100X, 1000X, and 10,000X (5 × 10^2^ PFU/mL) in human urine for extraction (100 µL per extraction) and detection. These levels of ZIKV in urine are clinically relevant [34,35,36,37]. RNA was extracted in quadruplicate using the NucliSENS Magnetic Particle Extraction Kit (bioMérieux, Durham, NC, USA) either manually or automatically with the VSPP device. The RNA template extracted using the VSPP device was evaluated in a commercial real-time PCR detection system (Bio-Rad CFX-96, Hercules, CA, USA) using a previously reported RT-PCR assay and thermal cycling conditions [23].

### 2.4. DNA Isolation from Human Whole Blood

We performed extraction from 80 μL whole blood samples to test the ability of the VSPP to handle complex samples. As a control, blood samples were extracted using a Promega Maxwell device with the Promega Maxwell RSC Whole Blood DNA Kit (AS1520, Promega, Madison, WI, USA). The extraction of human DNA was carried out using real-time PCR with RNase P primers. We increased the magnetic bead resuspension during the washing and elution steps by alternating the magnetic forces in our cartridge. Anonymized whole blood was purchased from Research Blood Components, LLC (Brighton, MA, USA).

### 2.5. Demonstrating That the VSPP’s Mechanical Operation Is Gravity-Independent

We first conducted tests to demonstrate that the basic mechanical function could operate independently of the device’s physical orientation against gravity by orienting the VSPP upside-down while in operation. This work was first performed without the cartridge or fluids. Next, food coloring dye was added to the wells and the VSPP was oriented sideways. The dye was added to increase the liquid’s visibility and aid in monitoring how well the liquid remained in the wells when the orientation of the VSPP was changed.

**Figure 4:**
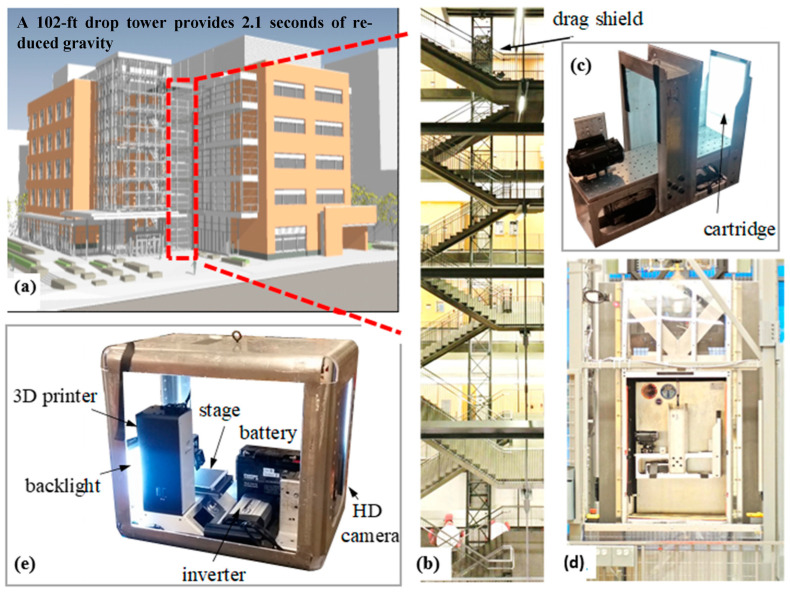
Drop tower at Portland State University that provides reduced gravity during a free fall. (**a**) PSU engineering building. (**b**) The six-story, 2.1 s Dryden Drop Tower. (**c**) Typical drop tower rig fitted for the fluid cartridge tests to note fluid reorientation and stability during gravity transition. (**d**) The rig installed in a drag shield prior to drop. (**e**) A rig that houses the equipment and camera. A back light allows the camera to capture better pictures of the fluidic motion.

To demonstrate the potential of the use of our device and cartridges in microgravity, we performed a series of tests at Portland State University’s Dryden Drop Tower (DDT) (Figure 4). Drop towers are structures used to produce a controlled period of weightlessness for an object under study by releasing it in a state of freefall. The DDT is a 102 ft-tall structure that provides 2.1 s of reduced gravity, suitable for the initial testing of the effects of microgravity on our device. During a two-day period, we performed over 30 drops. High-speed videos and photos were taken using the setup and analyzed to determine if our setup would be compatible with microgravity. In addition, we learned that our hardware setup is robust enough to be tested with repeated drops and sudden stops in the drop tower.

### 2.6. Impact of 1 g and Low g on Various Test Fluids

We aimed to test how different pertinent test fluids transition between 1 g and low g states in cartridges and wells cut from 96-well DNA extraction plates (VWR 82007-292 Disposable 96 Deep Well Plate, 1.2 mL). The test fluids chosen are as follows: NucliSENS lysis buffer (bioMérieux, Durham, NC, USA) with nucleic acid-binding magnetic particles, NucliSENS wash buffer 1, NucliSENS wash buffer 2, NucliSENS wash buffer 3, NucliSENS elution buffer, Dynabeads lysis buffer (Dynabeads^®^ SILANE Viral NA Kit, Invitrogen, Carlsbad, CA, USA) with nucleic acid-binding magnetic particles, Dynabeads wash buffer 1, and Dynabeads wash buffer 2. The reagents were added to the wells and exposed to brief microgravity during the drops. Wells with significant capillary corner wicking were identified.

### 2.7. Testing for Generation and Contamination from Satellite Droplets 

We tested the severity of satellite droplets by inserting and withdrawing a 7-millimeter-wide rounded polypropylene extraction tip from wells containing different types of solution and studying if moving the extraction tip between wells could lead to cross-contamination issues from satellite droplets. Drop tower tests were performed with the extraction tip repeatedly inserted and withdrawn from cylindrical and the 3D printed tapered rhombic wells containing various liquids (i.e., water, buffer solutions, etc.). 

### 2.8. Optimizing the Fluidic Control of Reagent Wells in Microgravity

To ensure that the fluids could be manipulated without spillage in the enclosed cartridges, we explored different avenues of fluidic control, including creating microgravity-compatible reagent wells. The wells’ geometry was designed to ensure that the reagents remain inside the wells regardless of the ISS’s direction of gravity or movement. Such geometries would ideally need to target a broad range of fluid types, including lysis, wash, and elution buffers. Our design was based on results previously obtained during ISS capillary fluidics experiments. These experiments demonstrated that tapered rhombic geometry promotes wetting and passively separates and migrates bubbles to the lid [38,39]. This forces the bubbles out of the liquid and leaves the liquid behind for terrestrial-like operations. The proliferation of low-cost 3D printers and 3D printing technologies has eased and expedited the process to design, test, and make improvements to designs via 3D printing, including the prototypes we tested in the drop towers.

## 3. Results

### 3.1. Simultaneous Sample Preparation with Magnetic Coupling

One of the advantages of our device is the ability to simultaneously automate sample preparation on multiple samples to save crew time and improve the consistency of the results. We tested different actuation speeds with the tip-comb to discern the maximum speed before the coupled magnets would become dislodged (see Figure 2b). We were able to use tip-comb movements as fast as 3000 mm/min (5 cm/s) without disrupting the connection to the inside extraction tips in the six cartridges. This also showed that the VSPP had successfully synchronized rapid movements within multiple enclosed cartridges to process nucleic acid isolation in parallel. The synchronized movement of the magnetic tip-combs created highly reproducible and controllable movements inside each cartridge for sample processing. Since the VSPP is based on a 3D printer, the magnet motion can be easily reprogrammed to accommodate a different number or shape of wells. Our cartridge design can be scaled to different volumes as desired by NASA and potential end-users.

### 3.2. The VSPP Operation Is As Reproducible As the Manual Operation

**Figure 5:**
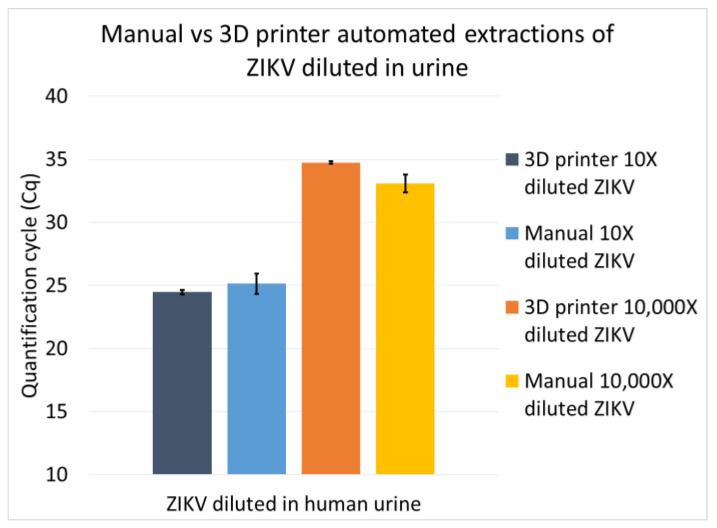
Extraction of nucleic acids from human urine performed manually vs. automated using a 3D-printer-inspired VSPP. Two concentrations of ZIKV were spiked in urine. Each extraction had four replicates (*n* = 4).

Next, extractions were performed to show that the automated VSPP could produce results comparable to those obtained via manual operation. The Zika virus culture was diluted to 10X or 10,000X in human urine and RNA was extracted in quadruplicate manually and automatically by our modified VSPP device. Overall, the manual extractions had a larger variation in yield based on the real-time RT-PCR quantification cycle (Cq) values (Figure 5). For the 10X diluted samples, the Cq values from both the manual (25.14 ± 0.80 standard deviation) and VSPP (24.46 ± 0.18) methods were essentially the same. For the 10,000X-diluted samples, the Cq values obtained using the manual method (34.08 ± 0.71) were only slightly lower than those found using the VSPP method (34.74 ± 0.08), suggesting that there was a slightly higher concentration of template recovered. We speculate that improving the efficiency of the heated elution on the VSPP can improve the yield at very low concentrations (e.g., Cq > 30). These results show that our automatic protocol using the VSPP worked relatively well compared to manual extractions and can be improved as we further optimize the automated extraction protocol. 

### 3.3. The VSPP Can Consistently Process a Wide Range of Target Concentrations

**Figure 6:**
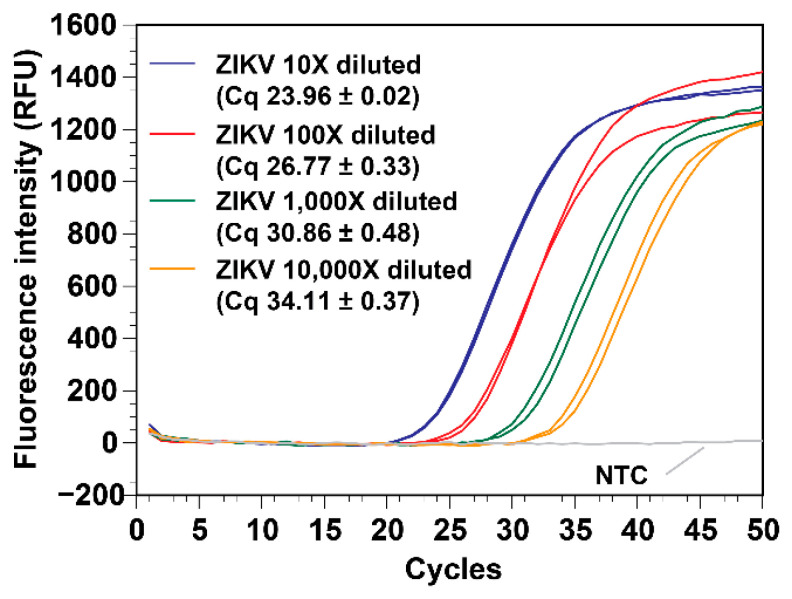
A 3D printer turned VSPP can extract a wide range of RNA from Zika virus spiked in human urine from 10X- to 10,000X-diluted samples (*n* = 2). NTC = no template control (*n* = 1).

To test the VSPP’s range, the Zika virus sample was serially diluted (10X, 100X, 1000X, and 10,000X) in human urine. The RNA was extracted in duplicate using the VSPP. The real-time RT-PCR results (Figure 6) showed that the RNA yield of all the concentrations was consistent with the expected values. The R^2^ value of the Cq vs. concentration plot was 0.991 and the slope was −3.45 (not shown), indicating that the extraction and real-time RT-PCR were highly efficient at the RNA concentration range tested.

### 3.4. Demonstrate DNA Isolation from Human Whole Blood

**Figure 7:**
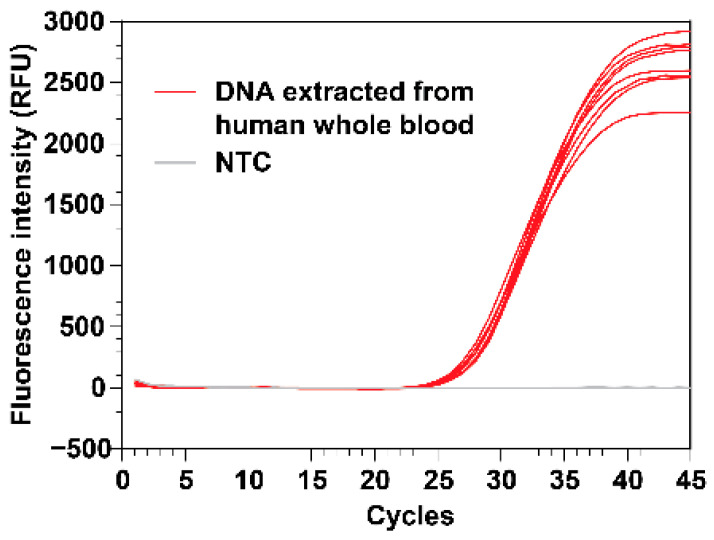
Extraction of replicate samples in parallel. Real-time PCR detecting the human RNase P gene, using DNA templates extracted from human whole blood. Mean Cq = 27.5, standard deviation = 0.4. The real-time curves suggested that the yield from the replicate samples (*n* = 8) was similar. A no-template control (*n* = 1) was added in the same PCR run.

To challenge our system for complex samples, we extracted DNA from human whole blood specimens. These real-time PCR experiments using RNase P as the gene target showed that the yield of human DNA from whole blood was consistent across eight extractions (mean Cq 27.5, SD 0.4) (Figure 7). We also performed parallel DNA extractions using the commercially available Promega Maxwell automated system. The RNase P real-time PCR results indicated that the yield of human whole blood DNA extracted using our VSPP is comparable to the yield extracted using Promega Maxwell (mean Cq 26.0, SD 0.5, *n* = 4). We suspect the higher yield (as determined by the lower Cq values from the real-time reaction) obtained from the Promega Maxwell was partly due to its more effective heating of the DNA elution. We could improve the elution step in our VSPP device by developing an improved heater block to increase the contact with the bottom of the polypropylene cartridge well.

### 3.5. Gravity-Independent Mechanical Operation

**Figure 8:**
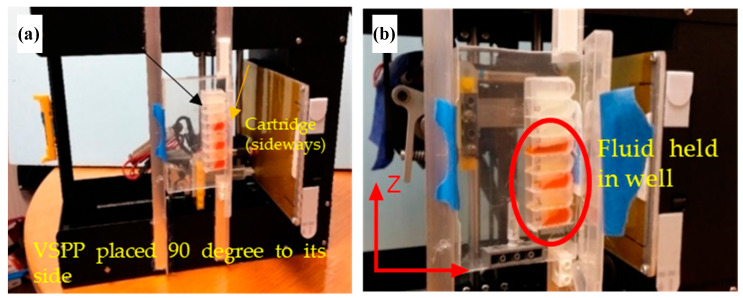
Modified orientation tests in a ground level lab. (**a**) Picture of the VSPP rotated 90° with the cartridge filled with food coloring fluid. The colored fluid in the cartridge well was held by surface tension and adhesion from the well walls and did not spill out. (**b**) Close-up of the cartridge showing that some fluid was contained because of surface tension.

To test if the hardware would function well in microgravity, the VSPP (modified from a Printrbot Play 3D printer in this case) was operated while upside down to test its ability to operate despite orientation without reduced speeds or dislodged parts. This work was first performed without the cartridge and fluids. After a successful operation, Figure 8 shows that the VSPP can also function when rotated 90 degrees sideways, even with fluid inside the wells. This work showed that, mechanically, the device could perform regardless of its orientation against gravity.

**Figure 9:**
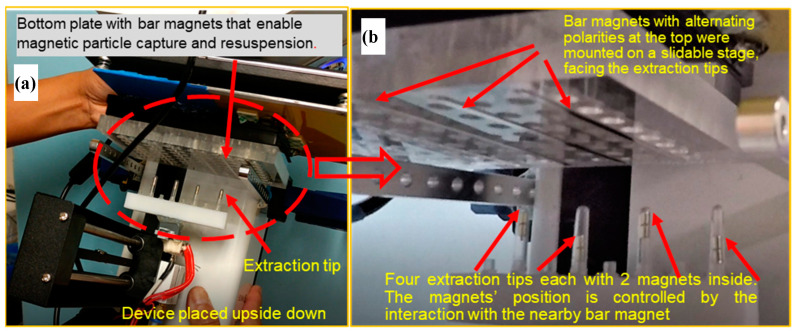
(**a**) Mechanical operation of a 3D printer turned VSPP while being held upside down. The magnets (red arrows) can be used to control the efforts of magnetic particle binding and washing. (**b**) The magnet position inside an extraction tip can be manipulated by the precise positioning of a set of bar magnets with opposite fields on the top side to attract or repel the magnets inside the extraction tip. The position of the magnets will enable the mixing of nucleic acid-binding magnetic particles by pulling the particles to the tip or the bottom of a reagent well.

We next tested whether we could fully control the position of the magnets inside the extraction tip-comb while operating the VSPP upside down (Figure 9). The VSPP in this work was modified from a Monoprice Select Mini 3D printer that cost USD 200. This work was first performed with empty wells, as it is difficult to keep the fluids from dripping out of the wells in 1 g conditions. Later, the use of a very small volume of fluid (e.g., 50 µL) allowed the adhesion force to keep the fluid inside a well when placed upside down. The magnets inside each extraction tip were raised or lowered when exposed to the magnetic field of a set of bar magnets (with opposite magnetic field at the top side) contained inside a plexiglass block, which were slid horizontally in and out of range of the extraction magnet tips as controlled via a stepper motor. The opposite field of the bar magnets was used to ensure that the position of the small magnet inside the tips could be controlled to enable the mixing of magnetic particles by pulling the particles to the tip or the bottom of the reagent well [29]. 

Our results indicated that the mechanical operation could still suspend and wash magnetic particles even when the VSPP was oriented completely upside down. This suggests that high-quality nucleic acid extraction with magnetic particle washing can be performed in microgravity. The effectiveness of this operation in microgravity will need to be tested in extended low g conditions.

### 3.6. Drop Tower Testing

#### 3.6.1. Extraction Well Geometry and Design

**Figure 10:**
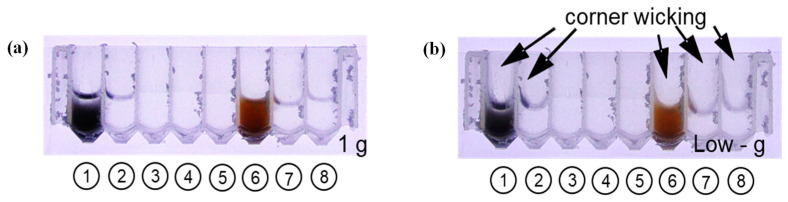
1 g (**a**) and low g (**b**) interface configurations in an 8-well cartridge: (left to right) NucliSENS lysis buffer with nucleic acid-binding magnetic particles (well #1), NucliSENS wash buffer 1 (well #2), NucliSENS wash buffer 2 (well #3), NucliSENS wash buffer 3 (well #4), NucliSENS elution buffer (well #5), Dynabeads lysis buffer with nucleic acid-binding magnetic particles (well # 6), Dynabeads wash buffer 1 (well #7), and Dynabeads wash buffer 2 well #(8). Wells with significant capillary corner wicking in low g were identified via drop tower tests.

The material and geometry of liquid-bearing wells are critical for the passive control of liquid in the absence of gravity. Without this control, liquids may not be accessible due to unexpected configurations and the presence of rogue bubbles. The drop tower rig shown in Figure 4 was used to test how the well plate geometry was affected by microgravity. For this set of experiments, NucliSENS reagents were used during the extraction process. Figure 10 presents the 1 g and low g configurations of a sample-loaded well cartridge where various corner wicking events occurred. Corner wicking was more severe in wells with certain liquids, such as the lysis buffer with magnetic particles for both vendors (NucliSENS wash buffer #1 and both Dynabead wash buffers). These findings will aid future work when considering the wicking effect as we design reagent wells to minimize these effects. 

**Figure 11:**
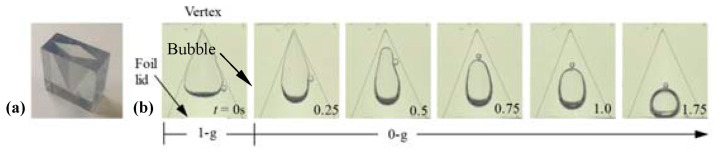
3D printed well design and drop tower test demonstration. (**a**) A 3D printed sample well for passive liquid control during low g fluidics sample preparation demonstrations. (**b**) A drop tower test of the worst-case 1 g inverted-sample well demonstrates passive bubble migration in a wetting buffer solution to the desired location, a centered-over-lid configuration. Time (t) shows bubble movement from 0 to 1.75 s.

To minimize the wicking effect in the 1 g extraction setup, modified reagent wells were designed to determine if they would be suitable for containing extraction reagents under microgravity. A transparent stereolithography (SLA) 3D printed well is shown in Figure 11a. As previously demonstrated during ISS capillary fluidics experiments, tapered rhombic geometry promotes wetting by passively separating and migrating bubbles to the lid [38,40]. Such geometries target a broad range of fluid types, including lysis, wash, elution buffer, detergents, clean water, etc. 

The drop tower test shown in Figure 11b demonstrates this phenomenon, as the bubbles passively migrated from a worst-case vertex position in 1 g to a centered-over-lid location in low g in the upside-down well. This centered-over-lid location is ideal for lid removal and subsequent sample preparation operations. Fortunately for the VSPP, the sample well design calculations can ensure stability during crew-induced perturbations. As the fluid volumes desired for extraction are small, this enabled a full-scale system to be adequately studied in the brief 1 g and low g conditions during the drop tower tests. Additional low g aircraft experiments can also be further designed to demonstrate that the wells and liquids were relatively insensitive to the g-perturbations of the aircraft. 

#### 3.6.2. Extraction Tip Insertion and Withdrawal Test

During the extraction procedure, extraction tips need to be inserted and withdrawn from different fluids in different wells. However, these movements may lead to bubble ingestion and rupturing films, leading to spurious satellite drops and liquid hold-ups. Though this may cause some concerns during terrestrial applications, the presence of gravity ensures that free bubbles rise and free drops fall. This is not the case for such operations in low g environments. We tested different speeds of extraction tip movement into 3D printed wells containing various fluids. In general, the low g interface was stable and, provided that the tip motions were slow and the fluid properties were wetting and viscous, no bubbles were created during insertion and no observable satellite drops were created or identified during withdrawal. 

**Figure 12:**
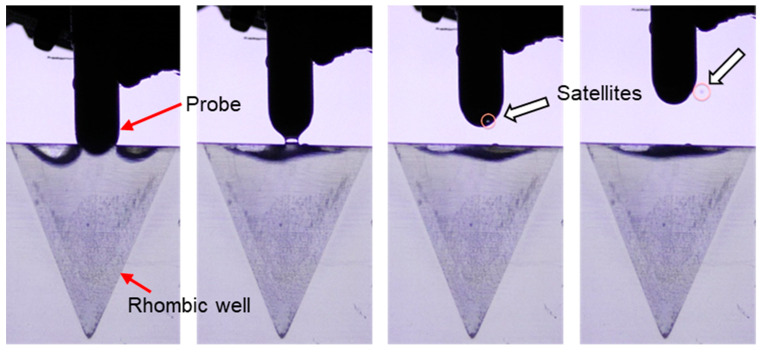
A 7 mm rounded polypropylene probe withdrawn from a 3D printed tapered rhombic well in a drop tower experiment at 50 mm/s. Satellite droplets of 0.5 mm diameter were observed (arrow) with similar speeds and trajectories following every withdrawal, indicating a poor geometry of probe for such operations was chosen. Future development efforts will be made to eliminate or minimize the escape of these satellite droplets.

However, spurious bubbles and drops were readily observed for various motions, probe tip and well geometries, and fluid types if the movements were fast. For example, in Figure 12, during each withdrawal step from water, satellite droplets measuring <0.5 mm in diameter were observed with similar speeds and trajectories, suggesting that poor choices of probe tip geometry, wetting conditions, and probe withdrawal rates can lead to the generation of satellite droplets. However, these conditions are likely avoidable and can be identified in future research and development efforts.

Although the satellite droplets are minuscule, it is vital to recognize the risk that such drops can lead to unwanted cross-contamination between wells within the cartridge. Special considerations for capillary controls for the extraction tip’s wetting and geometry, well-wetting geometry, and control patterns (e.g., acceleration and velocity vectors) can significantly mitigate these potential ill-effects between wells and within each cartridge. However, as we are developing self-contained cartridges for individual samples, the satellite droplets will not cause any cross-contamination between cartridges. If additional containment is needed, work can also be completed inside the Microgravity Sciences Glovebox (MSG) or a disposable glove bag to minimize sample transfer or contamination concerns. As shown in our results, it appears that the current low g capillary fluidics knowledge is adequate to produce safe, reliable, and elegant sample preparation fluid operations in microgravity. 

#### 3.6.3. Microgravity Cartridge Design and Prototype

**Figure 13:**
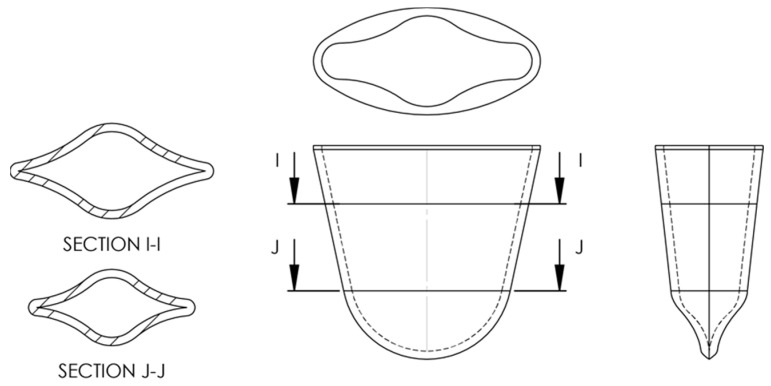
The well (~1 mL) is designed to accommodate a probe, preferentially locate fluid toward the bottom of the well, and inhibit wicking near the top of the well. For optimal performance, the upper portion of the well and lid (presumably a thin aluminum or PTFE film) should be non-wetting to discourage the development of wall bound drops and adverse wetting.

A microgravity-compatible well (Figure 13) was developed based on the information obtained and illustrated in Figure 10, Figure 11 and Figure 12. Connecting multiples of these wells together can form an extraction cartridge. The well, with a capacity of 1 mL, was designed to accommodate an extraction tip, preferentially locate wetting liquids to the base, and discourage wetting the upper portion of the well, which should be hydrophobic. This prevents volume loss from the bottom of the well and non-ideal fluid location during film puncturing. Hydrophobicity may be achieved by using a treated foil film or polytetrafluoroethylene (PTFE). Due to the variation in wetting, the well has a sharp corner to promote wicking for poorly wetting fluids. The corner becomes more acute as it moves toward the base of the well and terminates in a sharp cusp. At the top of the well, the corners are rounded to discourage wicking near the lid. This means that if the foil cover is removed, the chances of fluid escaping will be minimized.

#### 3.6.4. Cartridge and Reagent Wells 3D Printed and Tested

**Figure 14:**
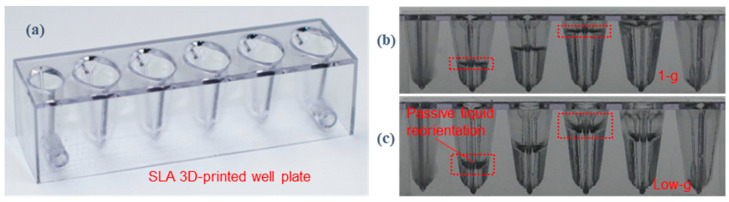
A 3D printed sample well plate design for use in 1 g and low g environments. (**a**) A sample well plate was designed to demonstrate well volume, injection, withdrawal, and low g performance. Drop tower tests for perfectly wetting polydimethylsiloxane oil were performed and are shown in (**b**) 1 g and (**c**) low g conditions. Fluid volume was 200, 400, 800, and 900 µL for the four middle wells (from left to right). Passive fluid reorientation was observed in all four filled wells during the 2.1 s of free fall. We note that only two of the four wells were marked to enable clear viewing of the wells and the fluid. Fluid performance at different fill levels in the wells was determined to be favorable for microgravity use as the fluid stayed in the desired position in the wells during the free fall.

With an individual well design in hand, a well plate with different containment volumes was then generated using SLA 3D printing (Figure 14). The plate incorporates the same design aspects as the individual well and includes ports for the injection and withdrawal of a sample. The plate was tested in low g using a drop tower. In general, highly wetting fluids quickly reoriented to the preferred location, while poorly wetting fluids, such as water, wicked more slowly. It is expected that, given enough time (i.e., being stored aboard the ISS), the poorly wetting fluids would eventually reorient to the preferred location. One may consider material selection or the use of hydrophilic coatings to enhance wetting in order to obtain a faster response or enhanced fluidic control.

## 4. Discussion

We described our expanded work on the development of a microgravity-compatible nucleic acid extraction device using low-cost 3D printers. Furthermore, 3D printing technologies also enabled us to quickly design, 3D print, and test the microgravity-compatible wells and cartridge. To ensure its compatibility in space, we also tested the VSPP’s ability to mechanically function in various orientations, such as upside down without fluids and sideways with fluids. Our ground-level laboratory tests proved that the VSPP could simultaneously process multiple samples with the tip-comb moving at speeds of up to 5 cm/s. Its operation yields results comparable to those of manual operations, even with a wide range of tested concentrations. The extraction and purification of DNA and RNA were performed to demonstrate that our low-cost 3D printer-based VSPP can conduct extractions and produce results with high consistency. This also demonstrated that mixing and washing magnetic particles could be integrated into our process. Additionally, whole blood samples were tested to ensure that complex samples could be successfully used with the VSPP.

The VSPP’s major components underwent several successful preliminary drop tower tests. These tests revealed the general aspects of fluidic control that need to be addressed as we move forward with extensively testing the device and cartridge in more prolonged low g conditions, such as parabolic flights or Blue Origin New Shepard suborbital rocket flights. Our development of a microgravity-compatible well using passive control will be of value for a wide variety of sample-processing steps for NASA and the larger aerospace community [41]. 

In conclusion, 3D printers and 3D printing technologies have allowed us to use a relatively low-cost and fast approach to prototype the VSPP and the DNA/RNA extraction cartridge. Our results show that the VSPP is a promising, viable solution for sample preparation procedures in zero g or low g operations in space. This manuscript was devised to provide preliminary information about our approach to developing a tool for future use on the ISS. The successful development of a flight-ready device will require substantial funding and the opportunity to test the device in suborbital and then orbital conditions. The information presented here by no means suggests that the VSPP is ready to fly the device to the ISS. However, we are confident that the current hardware is safe and simple to operate in space by the crew. The self-contained, enclosed cartridge design will act as the primary containment layer to prevent leakage that can present dangers to the ISS crew. We next need to build and validate the entire protocol in extended reduced-gravity conditions and present the findings of testing in parabolic flights and suborbital flights. We are working toward securing additional NASA resources to accomplish these research and development activities.

## Data Availability

The data presented in this study are available in the article.
